# 

**Published:** 1820-11

**Authors:** 


### MONTHLY CATALOGUE OF MEDICAL BOOKS.

Outlines of Midwifery, developing its Principles and Practice; with twelve Lithographic Engravings. By J. T. Conquest, M.D. F.L.S. &c. 7s. 6d.

A Toxicological Chart; in which may be seen, at one view, the Symptoms, Treatment, and Mode of Detecting, the various Poisons, Mineral, Vegetable, and Animal, according to the latest Experiments and Observations. Respectfully dedicated to the Royal Humane Society. By Wm. Stowe, Member of the London Royal College of Surgeons. The Second Edition, with Additions and Improvements. On two large Sheets, broad folio, 1s. 6d.; or, neatly mounted on a board, 2s. 6d.

A Catalogue of Second-hand Medical Books on sale by John Anderson, Medical Bookseller, 40, West Smithfield.

A Practical Treatise on those Nervous Diseases which are denominated Hypochondriasis, or Low Spirits, and Dyspepsia, or Indigestion: illustrated with numerous Cases, exhibiting the most successful mode of Treatment; equally addressed to Invalids as to those of the Profession. By George Robert Rowe, Fellow of the Royal College of Surgeons, London, &c. 8vo. 6s.

Medico-Chirurgical Transactions, by the Medico and Chirurgical Society of London. Vol. II. Part I. 8vo. 9s.

Examinations in Anatomy, Physiology, Practice of Physic, Surgery, Chemistry, Materia Medica, and Pharmacy, for the use of Students. By Robert Hooper, M.D. Fourth Edition, greatly enlarged and improved. 12mo. 5s. 6d.

A Sketch of the History and Cure of Febrile Diseases; more particularly as they appear in the West Indies among the Soldiers of the British Army. By Robert Jackson, M.D. Second Edition, with many Additions. 2 vols. 8vo. 1l. 4s.

A Chemical and Medical Report of the Properties of the Mineral Waters of Buxton, Matlock, Tunbridge Wells, Harrogate, Bath, Cheltenham, Leamington, Malvern, and the Isle of Wight. By Charles Scudamore, M.D. 8vo. 10s.

[MESSRS. HIGHLEY AND SON.]

